# Primary apocrine sweat gland carcinomas of the axilla: a report of two cases and a review of the literature

**DOI:** 10.1186/s12957-015-0473-1

**Published:** 2015-02-17

**Authors:** Min‐Ki Seong, Eun-Kyu Kim, Kanghee Han, Hyesil Seol, Hyun‐Ah Kim, Woo Chul Noh

**Affiliations:** Department of Surgery, Korea Cancer Center Hospital, Korea Institute of Radiological and Medical Sciences, 215‐4 Gongneung‐dong, 139-706 Nowon‐ku, Seoul Republic of Korea; Department of Pathology, Korea Cancer Center Hospital, Korea Institute of Radiological and Medical Sciences, 215‐4 Gongneung‐dong, 139-706 Nowon‐ku, Seoul Republic of Korea; Department of Surgery, Breast Cancer Center, Seoul National University Bundang Hospital, Seoul National University College of Medicine, 82, Gumi-ro 173 beon-gil, Bundang-gu, Seongnam-si, Gyeonggi-do 463-707 Republic of Korea

**Keywords:** Primary apocrine sweat gland carcinoma, Clinicopathological features, Diagnosis, Treatment guidelines

## Abstract

Primary apocrine sweat gland carcinoma (PASGC) is an extremely rare malignancy with a relatively favorable prognosis. PASGC is often suspected to be a benign disease during an initial clinical examination, which leads to inadequate initial treatment and extensive metastasis. Owing to the limited number of reports on PASGC, its diagnostic criteria and treatment guidelines have not yet been established. The only known curative therapy for localized PASGC is wide local excision. In the present report, we describe two cases of PASGC with locally aggressive disease that arose in the axilla and review the literature about its clinicopathological features, diagnosis, and treatment. Based on the findings of the current report, we suggest that a sentinel lymph node biopsy and adjuvant anti-estrogen therapy should be included in the management of PASGC.

## Background

Primary apocrine sweat gland carcinoma (PASGC) is a rare subtype of sweat gland carcinoma, with approximately 50 cases reported in the literature thus far [1-5]. It occurs mostly in apocrine-dense regions such as the axilla and anogenital areas, although it has also been reported to occur in less typical locations such as the forehead, wrists, ear canals, eyelids, trunk, feet, toes, and fingers [1-6]. This disease presents in older patients, with a median age at presentation of 67 years. No racial or gender differences in PASGC incidence have been reported. The incidence of PASGC is quite low at 0.0049 to 0.0173 cases/100,000 persons per year [1]. Most PASGCs are generally characterized by indolent symptomatic progression and a slow growth rate and are not clinically suspected prior to biopsy. Once the diagnosis is made, the treatment of choice for localized PASGC is wide local excision with a clear margin of 1 to 2 cm, along with axillary lymph node dissection if clinically positive nodes are detected [1-5,7]. The benefit of adjuvant chemotherapy for PASGC with or without lymph node metastases remains controversial. However, adjuvant radiotherapy may be recommended for locally advanced disease [7]. Although PASGC has a high incidence of local recurrence and regional lymph node metastasis, most patients have an indolent disease course. Only 14 cases of PASGC with distant metastasis to the lungs, liver, or bone have been reported to date [2]. In the largest retrospective cohort study of PASGC, the median overall survival and 5-year disease-specific survival rate were 51.5 months and 88%, respectively [1]. The prognostic factors for PASGC are difficult to identify because of the small number of reported cases, but the possible factors include tumor size, histological type, lymph node involvement, and distant metastasis [4,5]. Here, we report two cases of locally advanced PASGC that arose in the axilla, which is the most common site of PASGC. We also present a summary of the clinicopathological features, diagnosis, and treatment based on a review of the literature.

## Case presentation

### Case 1

**Figure 1:**
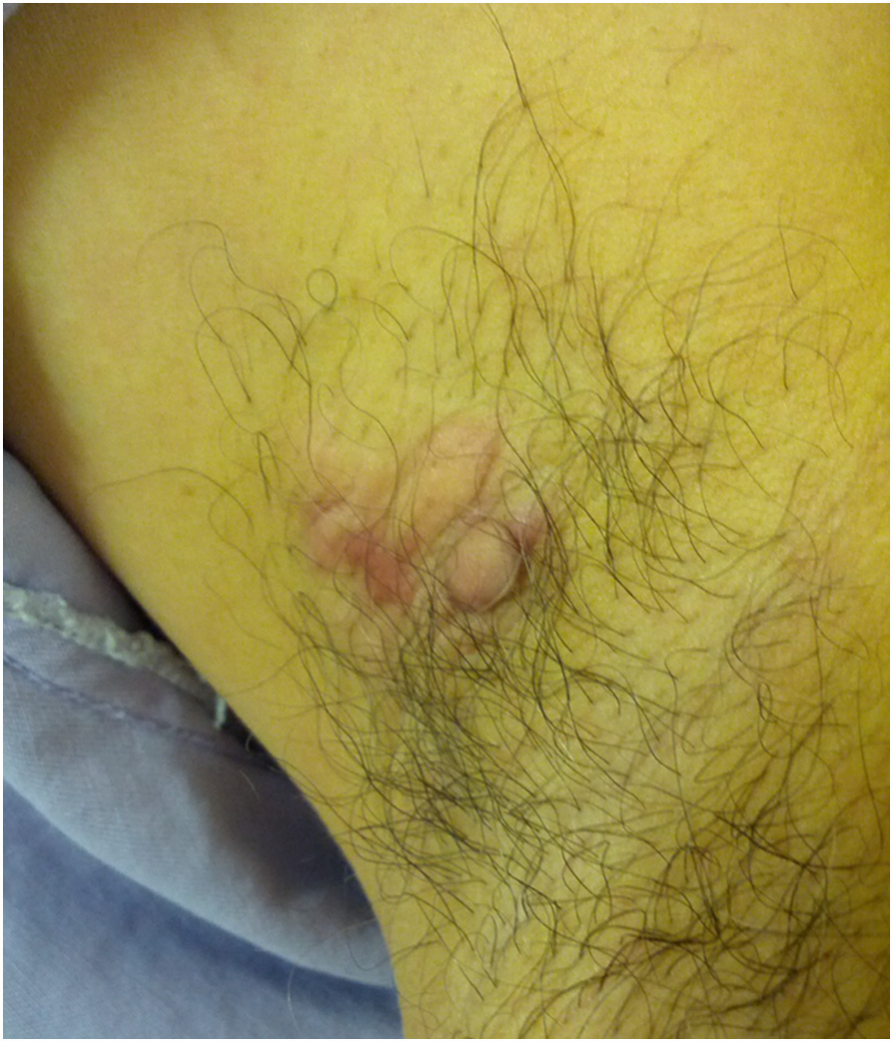
**A photograph of the left axillary mass prior to excision.** The mass, measuring 3 × 3 cm in size, was firm, erythematous, multilobular, indurated, and non-tender.

**Figure 2:**
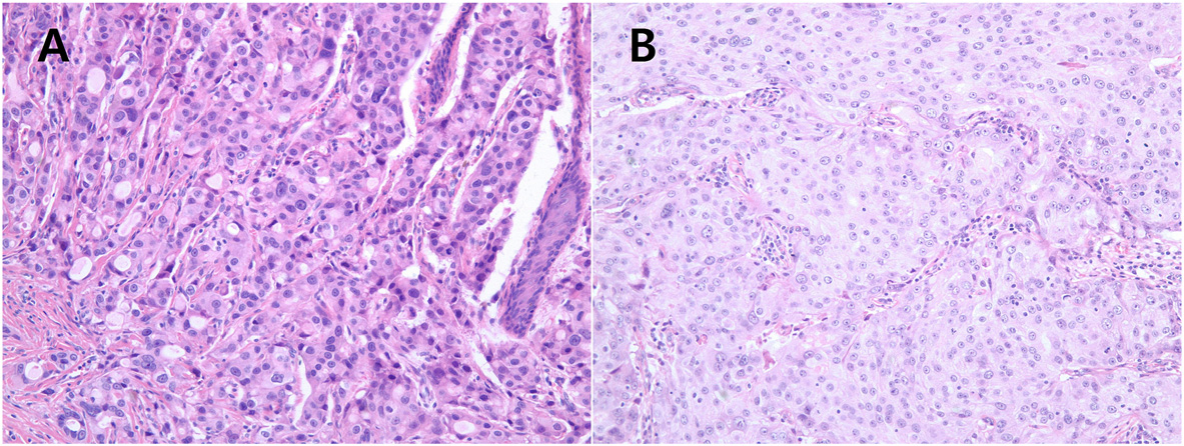
**Microscopic features of the tumor cells. (A, B)** The tumor cells contained abundant, granular, eosinophilic cytoplasm and enlarged nuclei with prominent nucleoli (hematoxylin and eosin stain, ×200).

**Figure 3:**
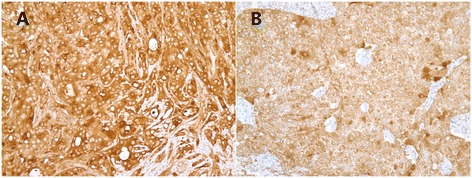
**Immunostaining for gross cystic disease fluid protein-15. (A, B)** Diffuse cytoplasmic staining of gross cystic disease fluid protein-15 was observed in the tumor cells (×200).

A 51-year-old man with a 5-mm-sized skin lesion in the right axilla was examined by a dermatologist in October 2011. The lesion was suspected to be an epidermal cyst (hidradenitis suppurativa). In December 2013, the patient was referred to the clinic of the Department of Surgery for excisional biopsy of the axillary mass, which had been growing rapidly over the previous 3 months. The patient had no symptoms or any medical or familial history of malignancy. On physical examination, a 4 × 3 cm-sized, erythematous, indurated, non-tender, and multilobular subcutaneous mass was palpated in the left axilla (Figure 1). Physical examination of the bilateral breast and contralateral axilla yielded unremarkable results. The mass was suspected to be a benign skin tumor and was completely excised and sent for histopathological examination. Macroscopic examination of the excised specimen revealed a 2 × 2 cm-sized tumor with a reddish colored cut surface. Microscopically, the malignant cells contained periodic acid-Schiff (PAS)-positive cytoplasmic granules, abundant eosinophilic cytoplasm, and apocrine-like decapitation (Figure 2A). On immunohistochemical staining, the tumor cells were positive for gross cystic disease fluid protein (GCDFP)-15 (Figure 3A), cytokeratin (CK) 7, and epithelial membrane antigen (EMA), and negative for CK20, S-100 protein, estrogen receptor (ER), and progesterone receptor (PR) (Table 1). Based on these findings, the preliminary pathological diagnosis was carcinoma with apocrine differentiation. To exclude other possible primary tumors, clinical evaluations including positron emission tomography-computed tomography (PET-CT), chest CT, abdominal and pelvic CT, colonoscopy, and duodenoscopy were performed, and no other occult potential primary site was identified. The diagnosis of PASGC was verified based on the pathologic features and the lack of any clinical evidence for a tumor originating from another site. PET CT and chest CT were performed 12 days after the initial excisional biopsy, and their findings were indicative of persistent disease in the right axilla. We therefore performed complete (including level III) axillary lymph node dissection and further excision of the involved margins. The final pathologic result included a 0.7 × 0.3 cm-sized residual carcinoma and metastasis in 50 dissected lymph nodes, including two level III lymph nodes. The patient underwent adjuvant radiation therapy to the right axilla and supraclavicular regions, with 50 Gy in 25 fractions over a period of 5 weeks. Radiation therapy was delivered using three-dimensional conformal radiotherapy with 10-MV photons. There was no evidence of recurrence 9 months after surgery.

**Table 1:** **Histological and immunohistochemical details of the two cases**

	**Case 1**	**Case 2**
PAS staining of diastase-resistant granules	Tumor cell focal (+)	Tumor cell focal (+)
	Secretion (+)	Secretion (+)
Eosinophilic cytoplasm	+	+
Decapitation secretion	+	+
GCDFP-15	+	+
CD15	+	+
CK7	+	+
CK20	−	+
Alpha-SMA	−	−
S-100	−	−
EMA	+	+
ER	−	−
PR	−	−
HER2	2+	2+
P53	Focal (+)	Focal (+)
CEA	−	Focal (+)

CEA, carcinoembryonic antigen; CK, cytokeratin; EMA, epithelial membrane antigen; ER, estrogen receptor; GCDFP-15, gross cystic disease fluid protein-15; HER2, human epidermal growth receptor 2; PAS, periodic acid-Schiff; PR, progesterone receptor; SMA, smooth muscle actin.

### Case 2

In November 2013, a 66-year-old man presented at our institution with a slowly growing, painful mass in the right axilla. The patient had been aware of the growing mass over the previous year, but the absence of pain had delayed his visit to the hospital. He had not experienced pain until just before the hospital visit. The patient had a medical history of endoscopic submucosal dissection for early gastric cancer 2 years previously, but no other medical or familial history of cancer. Physical examination revealed an approximately 4 × 3 cm-sized, firm, reddish, tender, indurated mass in the right axilla. No other lesions were found in the breasts or the contralateral axilla. Based on these clinical findings, a preliminary diagnosis of a benign skin tumor was considered, and surgical excision of the lesion was performed to determine the clinical diagnosis. Microscopically, the tumor cells contained enlarged nuclei with prominent nucleoli and PAS-positive granules and abundant eosinophilic cytoplasm with decapitation secretion (Figure 2B). Immunohistochemical staining revealed positive expression of GCDFP-15 (Figure 3B), CD15, CK7, CK20, and EMA (Table 1). These findings were consistent with those of carcinoma with apocrine differentiation. An extensive workup including PET-CT, chest CT, abdominal and pelvic CT, colonoscopy, and duodenoscopy did not reveal any other lesions suggestive of primary or secondary tumors, or any clinical evidence of recurrent or metastatic gastric cancer. Apocrine breast cancer was excluded on the basis of breast ultrasonography findings. After excluding the possibility of any other primary origin tumor through a complete clinical evaluation, a diagnosis of PASGC was made. As there was evidence of residual metastatic lymph nodes on the PET-CT and chest CT scans obtained 13 days after the initial excisional biopsy, an additional excision of the skin and subcutaneous tissue, along with axillary lymph node dissection, was performed to obtain microscopically clear margins. Axillary lymph node dissection was extended to the level III nodes. Pathologic examination revealed no residual carcinoma in the primary tumor site; however, all 12 resected axillary lymph nodes, including two level III lymph nodes, were found to contain metastases. The patient received adjuvant radiation therapy to the right axilla and supraclavicular regions, with a total dose of 50 Gy in 25 fractions over a period of 5 weeks. Radiation therapy was delivered using three-dimensional conformal radiotherapy with both 4 and 10 MV. The patient had no evidence of recurrence or metastasis during a 10-month follow-up.

## Conclusions

PASGC, a subtype of sweat gland carcinoma, is an extremely rare skin cancer [1-5]. Most cases of PASGC present with slowly enlarging, painless, colorless or reddish, indurated nodules or plaques. They are often initially misdiagnosed as benign skin tumors because of their indolent nature. In pathologic diagnosis, PASGC should be distinguished from other skin cancers, metastatic visceral adenocarcinomas, and breast carcinomas with apocrine features [3,6-8]. Definite diagnostic criteria for PASGC have not been established because of the limited number of cases. Although the diagnostic criteria for PASGC remain controversial, decapitation secretion in eosinophilic epithelial cells is considered a key indicator of apocrine differentiation [8]. In addition to this essential feature, Paties *et al.* proposed the following diagnostic criteria for PASGC: (1) decapitation secretion, (2) PAS-positive diastase-resistant material in the cells or lumina, and (3) positive immunostaining for GCDFP-15 (even if focal) [9]. A number of other reports have indicated that staining for CD15 and lysozyme may help distinguish between PASGC and eccrine carcinoma [10] and that androgen receptor positivity is strongly associated with PASGC carcinoma [11]. Thus, immunohistochemical findings could be helpful in diagnosing PASGC. However, given the lack of definite diagnostic criteria, any other possible primary origin must be ruled out through a full clinical workup before this malignancy can be definitely diagnosed [2,7,8]. The standard treatment for PASGC is wide local excision and regional lymph node dissection for clinically positive nodes. Despite the fact that regional lymph node metastases are found in nearly 50% of patients with PASGC at the time of diagnosis, the benefit of performing prophylactic regional lymph node dissection in patients with no clinical evidence of lymph node metastasis remains controversial. Some studies have shown that prophylactic lymph node dissection has no influence on survival or disease recurrence in PASGC patients [2,4]. However, several case reports have found that sentinel lymph node (SLN) biopsy is useful in these patients. As with other skin cancers, SLN biopsy may be helpful in assessing regional nodal status and decision-making regarding lymph node dissection in PASGC [12,13]. The role of postoperative adjuvant treatment in PASGC remains unclear. Adjuvant chemotherapy is not routinely offered, because PASGC is generally considered resistant to chemotherapy [3,7]. However, some case reports have described favorable responses to various chemotherapeutic agents in patients with metastatic PASGC [14-16], thus warranting further study. Compared with chemotherapy, radiotherapy may cure the disease or at least reduce the risk of relapse [7]. A marked response to radiation therapy with a total dose of 50 Gy in a patient with regional recurrence of PASGC has been reported [17]. Furthermore, Chamberlain *et al.* suggested that adjuvant radiotherapy should be considered if the disease has one or more of the following characteristics: large tumor size (≥5 cm), positive resection margin, moderately to poorly differentiated tumors, or vascular or lymphatic invasion [7]. Interestingly, PASGCs often express ER and PR, unlike most primary apocrine breast carcinomas [8]. The relatively frequent expression of these receptors may provide a rationale for anti-estrogen therapy, by using drugs such as tamoxifen. One case report described a complete response to tamoxifen in a patient with ER-positive metastatic PASGC [18]. In another case report, adjuvant tamoxifen therapy resulted in a disease-free survival of over 3 years after surgery for recurrent PASGC [19]. Therefore, anti-estrogen therapy may be considered not only for the treatment of metastatic PASGC but also for the adjuvant treatment of PASGC.

Because of its rarity, isolated case reports have been the main source of information regarding PASGC. Currently, there are no established guidelines for the management of this disease. Based on our literature review, we recommend the following for the treatment of localized PASGC: 1) a wide local excision with a 1- to 2-cm clear margin, 2) regional lymph node dissection for clinically positive nodes, 3) SLN for clinically negative nodes, 4) adjuvant radiation therapy (owing to the high local recurrence of this malignancy), and 5) adjuvant anti-estrogen therapy in patients with hormone receptor-positive tumors.

PASGC is a rare malignancy that arises from the sweat glands, for which diagnostic criteria and treatment guidelines have yet to be established. We reviewed the literature to identify clinicopathological characteristics that may help diagnose PASGC. We have also made a number of treatment recommendations for patients with PASGC, including the use of sentinel lymph node biopsy and adjuvant anti-estrogen therapy.

## Consent

Written informed consent was obtained from the patient for publication of this case report and any accompanying images. A copy of the written consent is available for review by the Editor-in-Chief of this journal.

## Abbreviations

CK: cytokeratin
CT: computed tomography
EMA: epithelial membrane antigen
GCDFP-15: gross cystic disease fluid protein-15
PAS: periodic acid-Schiff
PASGC: primary apocrine sweat gland carcinoma
PET: positron emission tomography
SLN: sentinel lymph node
